# RETRACTION: Effect of Serologic Malnutrition on Postoperative Wound Infection Problems After Total Joint Arthroplasty: A Meta‐Analysis

**DOI:** 10.1111/iwj.70460

**Published:** 2025-04-02

## Abstract

**RETRACTION:** ZhangD. and ZhangX. , “Effect of Serologic Malnutrition on Postoperative Wound Infection Problems After Total Joint Arthroplasty: A Meta‐Analysis,” International Wound Journal 20, no. 2 (2023): 261–268, 10.1111/iwj.13869.35833263 PMC9885483

The above article, published online on 14 July 2022, in Wiley Online Library (http://onlinelibrary.wiley.com/), has been retracted by agreement between the journal Editor in Chief, Professor Keith Harding; and John Wiley & Sons Ltd. Following an investigation by the publisher, all parties have concluded that this article was accepted solely on the basis of a compromised peer review process. The editors have therefore decided to retract the article. The authors did not respond to our notice regarding the retraction.

**RETRACTION:** D. Zhang and X. Zhang , “Effect of Serologic Malnutrition on Postoperative Wound Infection Problems After Total Joint Arthroplasty: A Meta‐Analysis,” International Wound Journal 20, no. 2 (2023): 261–268, 10.1111/iwj.13869.35833263 PMC9885483

The above article, published online on 14 July 2022, in Wiley Online Library (http://onlinelibrary.wiley.com/), has been retracted by agreement between the journal Editor in Chief, Professor Keith Harding; and John Wiley & Sons Ltd. Following an investigation by the publisher, all parties have concluded that this article was accepted solely on the basis of a compromised peer review process. The editors have therefore decided to retract the article. The authors did not respond to our notice regarding the retraction.

